# Studying real-world perceptual expertise

**DOI:** 10.3389/fpsyg.2014.00857

**Published:** 2014-08-06

**Authors:** Jianhong Shen, Michael L. Mack, Thomas J. Palmeri

**Affiliations:** ^1^ Vanderbilt Vision Research Center, Department of Psychology, Vanderbilt UniversityNashville, TN, USA; ^2^Center for Learning and Department of Psychology, The University of Texas at AustinAustin, TX, USA

**Keywords:** perceptual expertise, expertise, learning, categorization, recognition, birding

## Abstract

Significant insights into visual cognition have come from studying real-world perceptual expertise. Many have previously reviewed empirical findings and theoretical developments from this work. Here we instead provide a brief perspective on approaches, considerations, and challenges to studying real-world perceptual expertise. We discuss factors like choosing to use real-world versus artificial object domains of expertise, selecting a target domain of real-world perceptual expertise, recruiting experts, evaluating their level of expertise, and experimentally testing experts in the lab and online. Throughout our perspective, we highlight expert birding (also called birdwatching) as an example, as it has been used as a target domain for over two decades in the perceptual expertise literature.

## INTRODUCTION

In nearly every aspect of human endeavor, we find people who stand out for their high levels of skill and knowledge. We call them experts. Expertise has been studied in domains ranging from chess (Chase and Simon, 1973; Gobet and Charness, 2006; Connors and Campitelli, 2014; Leone et al., 2014) to physics (Chi et al., 1981) to sports (Baker et al., 2003). Perceptual experts, such as ornithologist, radiologists, and mycologists, are noted for their remarkable ability to rapidly and accurately recognize, categorize, and identify objects within some domain. Understanding the development of perceptual expertise is more than characterizing the behavior of individuals with uncanny abilities. Rather, if perceptual expertise is the endpoint of the trajectory of normal visual learning, then studying perceptual experts can provide insights into the general principles, limits, and possibilities of human learning and plasticity (e.g., Gauthier et al., 2010).

Several reviews have highlighted empirical findings and theoretical developments from research on perceptual expertise in various modalities (for visual expertise, see, e.g., McCandliss et al., 2003; Palmeri and Gauthier, 2004; Palmeri and Cottrell, 2009; Richler et al., 2011; for auditory expertise, see, e.g., Chartrand et al., 2008; Holt and Lotto, 2008; for tactile expertise, see, e.g., Behrmann and Ewell, 2003; Reuter et al., 2012). Here, we instead highlight more practical considerations that come with studying perceptual expertise; we highlight visual expertise because this modality has been most extensively studied. We specifically consider some choices that face researchers: whether to use real-world or artificial objects, what domain of perceptual expertise to study, how to recruit participants, how to evaluate their expertise, and whether to test in the lab or via the web. Throughout our perspective, we use birding as an example domain because it has been commonly used in the literature (e.g., Tanaka and Taylor, 1991; Gauthier et al., 2000; Tanaka et al., 2005; Mack et al., 2007; Mack and Palmeri, 2011).

## REAL-WORLD vs. ARTIFICIAL DOMAINS OF EXPERTISE

Expertise-related research has been conducted using both artificial and real-world objects. Artificial objects include simple stimuli like line orientations, textures, and colors (e.g., Goldstone, 1998; Mitchell and Hall, 2014), and relatively complex novel stimuli like random dot patterns (Palmeri, 1997), Greebles (Gauthier and Tarr, 1997; Gauthier et al., 1998, 1999), and Ziggerins (Wong et al., 2009a). Real-world objects include birds, dogs, cars, and other categories (Tanaka and Taylor, 1991; Gauthier et al., 2000). Studies using artificial objects are often training studies, where researchers recruit novices and train them to become “experts” in a domain. Changes in behavior or brain activity are measured over the course of training to understand the development of expertise, making these studies longitudinal. The weeks of training used in these studies can only be a proxy for the years of experience in real-world domains. Because real-world expertise takes so long to develop, most real-world studies are cross-sectional.

An advantage of training studies with artificial objects is the power to establish causality. Experimenters have precise control over properties of novel objects, relationships between them, and how categories are defined (e.g., Richler and Palmeri, 2014). Participants can be randomly assigned to conditions and training and testing can be carefully controlled. As one example, Wong et al. (2009a,b) used novel Ziggerins and trained people in two different ways, one of which mirrored individuation required for face recognition, another of which mirrored the letter recognition demands required for reading. Accordingly, the face-like training group showed behavior and brain activity similar to that seen in face recognition while the letter-like training group showed behavior and brain activity similar to that seen in letter recognition. Studies of artificial domains of expertise can provide insights into real-world domains.

If researchers are interested in understanding what makes experts experts, not just investigating limits of experience-related changes, then it is important to complement carefully controlled laboratory studies using artificial domains with the study of real-world experts. Because of their quasi-experimental nature – recruiting novices and those with varying levels of expertise as they occur in the real world – these studies cannot establish unambiguous causal relationships between expertise and behavioral or brain changes. Apart from considerations of external validity, studies of real-world experts permit the study of a range and extent of expertise that cannot easily be reproduced in the laboratory. And practically speaking, testing real-world perceptual experts on real-world perceptual stimuli saves researchers the effort and expense needed to train participants in an artificial domain.

**FIGURE 1:**
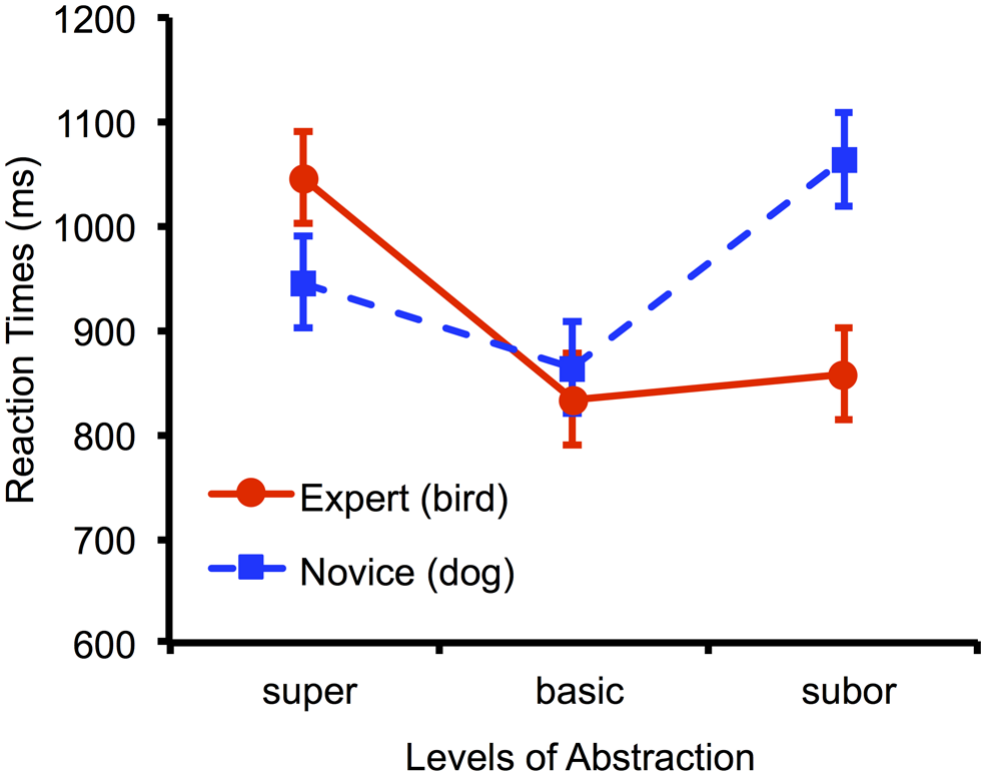
**Mean correct categorization response times for a novice domain (dogs) and an expert domain (birds) measured online.** Following Tanaka and Taylor (1991), bird experts were tested in a speeded category verification task where they categorized images at the superordinate (*animal*), basic (*bird* or *dog*), or subordinate (specific species or breed) level. In their novice domain (dogs), a classic basic-level advantage was observed, whereby categorization at the basic level was significantly faster than the superordinate (*t_22_* = 2.67, *p* = 0.014) and subordinate level (*t_22_* = 6.75, *p*< 0.001). In their expert domain (birds), subordinate categorization was as fast as basic-level categorization (*t_22_* = 0.81, *p* = 0.429). This replication was conducted using an online Wordpress + Flash custom website with only 23 participants from a single short 10 min experimental session. Error bars represent 95% confidence intervals on the level × domain interaction.

Studies using real-world domains also come full circle to inform studies using artificial domains. For example, consider the classic result of Tanaka and Taylor (1991), reproduced in our own online replication in **Figure 1**. Bird experts categorized birds (their expert domain) and dogs (their novice domain). For novices (Rosch et al., 1976), objects are categorized faster at a basic level (*dog*) than a superordinate (*animal*) or subordinate level (*blue jay*), while for experts (Tanaka and Taylor, 1991; Johnson and Mervis, 1997), objects are categorized as fast at a subordinate level as a basic level. This entry-level shift (Jolicoeur et al., 1984; see also Tanaka et al., 2005; Mack et al., 2009; Mack and Palmeri, 2011) has been used as a behavioral marker of expertise in training studies employing artificial domains (Gauthier et al., 2000; Gauthier and Tarr, 2002).

Our group recently reviewed considerations that factor into studies using artificial domains (Richler and Palmeri, 2014), so here we focus on real-world domains for the remainder of our perspective.

## DOMAINS OF REAL-WORLD PERCEPTUAL EXPERTISE

In addition to everyday domains of perceptual expertise, like faces (Bukach et al., 2006) and letters (McCandliss et al., 2003), studies have used domains ranging from cars and birds (Gauthier et al., 2000), where expertise is not uncommon, to more specialized and sometimes esoteric domains like latent fingerprint identification (Busey and Parada, 2010; Dror and Cole, 2010), budgie identification (Campbell and Tanaka, 2014), and chick sexing (Biederman and Shiffrar, 1987). The particular choice of expert domain depends on a combination of theoretical goals and practical considerations.

For example, consider a goal of understanding how the ability to categorize at different levels of abstraction changes with perceptual expertise (Mack and Palmeri, 2011), which impacts understanding of how categories are learned, represented, and accessed. Birding is a useful domain because birders must make subordinate and sub-subordinate categorizations, sometimes at a glance, and often under less than ideal conditions with poor lighting and camouflage. Other kinds of bird experts have different skills: budgie experts (a budgerigar is a bred parakeet) can keenly identify unique individuals in cages, but need not have expertise with other birds, while professional chick sexers can quickly discriminate male from female genitalia on chicken hatchlings. In an entirely different domain, fingerprint experts typically match latent prints with a known sample, with both clearly visible, presented side by side, and with time limits imposed by the analyst, not the environment.

There are real-world consequences for studying certain domains of perceptual expertise, such as latent fingerprint examination. Despite the widespread use of forensic evidence – as well as its popular depiction on television – a recent National Research Council of the National Academy of Sciences (2009) noted a “dearth of peer-reviewed, published studies establishing the scientific bases and validity of many forensic methods,” especially those methods that require subjective visual pattern analysis and expert testimony. That scientific evidence is emerging, especially in the case of latent fingerprint expertise (e.g., Busey and Parada, 2010; Busey and Dror, 2011).

The choice of domain can also be influenced by various practical considerations. It is easier to study perceptual expertise in a domain with millions of possible participants than an esoteric domain with a few isolated members. It is easier to study a domain where relevant stimuli are widely available in books and online. And it is easier to study a domain without barriers to contact, which can be the case for experts in the military, homeland security, and certain professions. For example, studies of expert baggage screeners require coordination with the Transportation Security Administration (TSA) and many details regarding stimuli and procedures cannot be shared with the public (e.g., Wolfe et al., 2013). In the case of birding, there are millions of people in the US alone who consider birding a hobby, spending hours in their yards and parks, and billions on books, equipment, and travel (La Rouche, 2006). Photos of birds are widely available; books have been published on particularly difficult bird identifications (e.g., Kaufman, 1999, 2011). Birders regularly participate in citizen science efforts, such as the Christmas bird count and provide data on bird sightings to databases like ebird.org. Anecdotally, this translates into a keen interest in science and a willingness to participate in research.

## RECRUITING

In the past, experts usually had to be recruited locally, with advertisements posted around a university campus and in local newspapers. It may be hard for some to remember that it has only been in the past several years that not having an email address has become almost equivalent to not having a phone number, and that only recently has it become the case that most people have some Internet access. Being able to recruit participants more widely via the Internet promises not only to increase heterogeneity of participants, but also, and especially relevant for expertise research, promises to locate participants with a far greater range of expertise than might be possible when recruiting in a local geographic region.

One rapidly exploding means of recruiting and testing (see “Testing”) participants is Amazon Mechanical Turk (AMT). AMT allows hundreds of subjects to be easily recruited and tested in a matter of days; participants on AMT are more demographically diverse than typical American college samples (Buhrmester et al., 2011). This diversity is important for research examining individual differences in perception and cognition. While the potential population of AMT workers is large, it is unknown how many with high levels of domain expertise might be workers on the platform. For expertise research, recruitment via AMT may need to be supplemented by more direct recruitment of true domain experts (e.g., Van Gulick, 2014).

Large domains of expertise have organizations, web sites, blogs, and even tweets and Facebook updates that target particular individuals. In principle, online recruiting through these channels offers a quick, easy, and inexpensive means of finding experts. These could involve paid advertisements online and in electronic newsletters. More directly, these could involve messages sent to email lists. The biggest challenge to this, however, is that many professional organizations or workplaces would rarely allow, and many outright prohibit, direct solicitation of members or employees, even for basic research; researchers cannot directly contact TSA baggage screeners or latent fingerprint examiners. By comparison, birding organizations, including local Ornithological and Audubon Societies, whose members join as part of a hobby, not a profession, can be less restrictive in terms of allowing contact with members, so long as contact is non-intrusive. In our case, we have identified several hundred birding groups in the US and Canada, we have contacted several dozen directly, and have received permission to solicit volunteer participants from most, having so far tested several hundred birders with a wide range of experience and expertise.

## EVALUATING LEVELS OF PERCEPTUAL EXPERTISE

How do we know someone is a perceptual expert? A simple approach relies on subjective self-rating, often supplemented by self-report on the amount of formal training, years of experience, or community reputation. For example, bird experts in Tanaka and Taylor (1991) were recommended by members of bird-watching organizations and had a minimum of 10 years of experience, and those in Johnson and Mervis (1997) led birding field trips and some had careers related to birding.

It is now well-recognized that self-reports of expertise are insufficient and that objective measures of expert performance are needed (e.g., Ericsson, 2006, 2009); self-report measures of perceptual expertise are not always good predictors of performance (e.g., McGugin et al., 2012; Van Gulick, 2014). Therefore, recent work has used quantitative measures to assess expert abilities (e.g., see Gauthier et al., 2010). A detailed review and discussion of such measures is well beyond the scope of a brief perspective piece. A variety of quantitative measures of perceptual expertise have been used and new measures are currently being developed – these efforts to develop and validate new measures reflect a quickly growing interest in exploring individual difference in visual cognition (e.g., Wilmer et al., 2010; Gauthier et al., 2013; Van Gulick, 2014).

While expert-novice differences are sometimes loosely described as if they were dichotomous, it is self-evident that expertise is a continuum, people vary in their level of expertise, and any measure of expertise must place individuals along a (perhaps multidimensional) continuum. Some behavioral or neural markers might distinguish pure novices from those with some experience but asymptote at only an intermediate level of expertise, while other behavioral or neural markers might distinguish the true experts from more middling experts and novices. Understanding the continuum of behavioral and brain changes, whether they are asymptotic, monotonic, or even non-monotonic over the continuum of expertise, can have important implications for understanding mechanistically and computationally how perceptual expertise develops (e.g., see Palmeri et al., 2004).

Briefly, one useful measure has focused on the perceptual part of perceptual expertise: using a simple one-back matching task, images are presented one at a time and participants must say whether consecutive pictures are the same or different. Experts have higher discriminability (d′) on images from their domain of expertise relative to non-expert domains, and this difference predicts behavioral and brain differences (e.g., Gauthier et al., 2000; Gauthier and Tarr, 2002). Another measure has focused on memory as an index of perceptual expertise: the Vanderbilt Expertise Task (VET; McGugin et al., 2012) mirrors aspects of the Cambridge Face Memory task (Duchaine and Nakayama, 2006). Participants memorize exemplars from several different artifact and natural categories and then recognize other instances under a variety of conditions, and these differences in memory within particular domains predict behavioral and brain differences (e.g., McGugin et al., 2014). With our interest in categorization at different levels of abstraction, in work in preparation, we have developed a measure that has focused on categorical knowledge in perceptual expertise: adapting common psychometric approaches, we are refining what could essentially be characterized as an Scholastic Assessment Test (SAT, a standardized test widely used for college admission in the United States) of birding knowledge, with multiple-choice identifications of bird images ranging from easy (common backyard birds like the Blue Jay), to intermediate (distinctive yet far less common birds, like the Pileated Woodpecker or Great Kiskadee), to quite difficult identifications that even fairly expert birders find difficult (like discriminating Bohemian from Cedar Waxwing, Hairy from Downy Woodpecker, or correctly identifying the many extremely similar warblers, sparrows, or flycatchers). Future work must consider to what extent different measures of perceptual expertise capture the same dimensions of expert knowledge and predict the same behavioral and brain measures that vary with expertise.

## TESTING

Laboratory testing allows careful control and monitoring of performance, permits experiments that require precisely-timed stimulus presentations, and of course allows sophisticated behavioral and brain measures like eye movements, fMRI, EEG, and the like. But laboratory testing incurs a potential cost in that the number of laboratory participants is often limited due to the expense of subject reimbursement, personnel hours, lab space, and equipment. And for any study of unique populations who might be geographically dispersed, such as perceptual experts, the cost of bringing participants to the laboratory can be prohibitively expensive.

Until fairly recently, the only real methods for testing participants from a wide geographic area, apart from having experimenters or participants travel, was to have the experiments travel. For simple studies, this could mean mailed pencil-and-paper tests, while for more sophisticated studies, this could mean sending disks or CDs to participants to run on a home computer (e.g., Tanaka et al., 2010). As anyone who programs well knows, getting software to run properly on a wide range of computer hardware and operating system versions can be a daunting task. In the past few years, it has become popular, and wildly successful, to have experiments run via a web browser. While not entirely immune to the vagaries of hardware and operating system versions, browser-based applications are often more robust to significant variation, and can often automatically prompt users for upgrades to requisite software plug-ins.

There are multiple platforms and approaches to online web-based experiments. One approach, highlighted earlier, uses AMT. In AMT, researchers publish Human Intelligence Tasks (HITs) that registered workers can complete in exchange for modest monetary compensation. AMT integrates low-level programming tools for stimulus creation, test design, and programming into one web-based application; other elements in AMT include automated compensation, recruitment, and data collection. Aside from the availability of these tools, a clear advantage of AMT is the potential to recruit from a large and diverse pool of participants. An alternative approach is to develop and support a custom web-based server for experiments. There are powerful tools for creating web pages, such as Wordpress (wordpress.org), and fairly sophisticated programs can be developed in Adobe Flash or Javascript (e.g., De Leeuw, 2014; Simcox and Fiez, 2014). Perhaps an advantage of such custom portals is that people may be more attracted to them because of their interest in participating in research, not because of the potential to earn money, as might sometimes be the case for AMT. In the end, we suspect that most labs will use a combination of both platforms for recruiting, testing, or both.

At least given current computer hardware in wide use, a potential vexing problem for web-based experiments is timing. Fortunately, platforms such as Flash and Javascript run on the local (participant) computer, so properly-designed programs can avoid problems that could be introduced by variability in Internet connection speeds. Thankfully, reasonable response time measurements can be obtained (Reimers and Stewart, 2007; Crump et al., 2013; Simcox and Fiez, 2014). Indeed, as illustrated in **Figure 1**, we have successfully observed differences in RTs for expert and novice domains in online experiments using a Wordpress + Flash environment that mirror observations of expert speeded categorization from classic laboratory studies (Tanaka and Taylor, 1991). Unfortunately, the most critical limitation for now concerns stimulus timing. It is well known that LCD monitors in wide use have response characteristics far too sluggish to permit the kind of “single-refresh” presentations that would have been possible on previous CRTs. While presentation times of 100 ms or more are probably a safe bet, anything faster would require calibration to check that a participant had a sufficiently responsive monitor; it may be that the next generation of LCD, LED, or other technologies will (hopefully) eliminate these limitations.

## SUMMARY

Most human endeavors have a perceptual component. For example, keen visual perception is required in sports, medicine, science, games like chess, and a wide range of skilled behavior. Thus research on real-world perceptual expertise has potential theoretical and applied impacts to many domains. Here we briefly outlined at least some of the practical considerations that factor into research on real-world perceptual expertise. Several of these considerations are things that researchers often fret over behind the scenes without making it into a typical research publication, so in that sense we hope this brief perspective fills a small but important hole in the literature.

## Conflict of Interest Statement

The authors declare that the research was conducted in the absence of any commercial or financial relationships that could be construed as a potential conflict of interest.

## References

[B1] Baker J. Cote J. Abernethy B. (2003). Sport-specific practice and the development of expert decision-making in team ball sports. *J. Appl. Sport Psychol.* 15 12–25 10.1080/10413200305400

[B2] Behrmann M. Ewell C. (2003). Expertise in tactile pattern recognition. *Psychol. Sci.* 14 480–492 10.1111/1467-9280.02458 12930480

[B3] Biederman I. Shiffrar M. M. (1987). Sexing day-old chicks: a case study and expert systems analysis of a difficult perceptual-learning task. *J. Exp. Psychol. Learn. Mem. Cogn.* 13 640–645 10.1037//0278-7393.13.4.640

[B4] Buhrmester M. Kwang T. Gosling S. D. (2011). Amazon’s Mechanical Turk: a new source of inexpensive, yet high-quality, data? *Perspect. Psychol. Sci.* 6 3–5 10.1177/1745691610393980 26162106

[B5] Bukach C. M. Gauthier I. Tarr M. J. (2006). Beyond faces and modularity: the power of an expertise framework. *Trends Cogn. Sci.* 10 159–166 10.1016/j.tics.2006.02.004 16516534

[B6] Busey T. A. Dror I. E. (2011). “Special abilities and vulnerabilities in forensic expertise,” in *Friction Ridge Sourcebook* ed. McRoberts A. (Washington, DC:NIJ Press) 1–23

[B7] Busey T. A. Parada F. J. (2010). The nature of expertise in fingerprint examiners. *Psychon. Bull. Rev.* 17 155–160 10.3758/PBR.17.2.155 20382913

[B8] Campbell A. Tanaka J. (2014). “Testing the face-specificity of the inversion effect in budgie experts,” in *Poster session presented at the Fourteenth Annual Meeting of the Vision Sciences Society* St. Pete Beach, FL.

[B9] Chartrand J. P. Peretz I. Belin P. (2008). Auditory recognition expertise and domain specificity. *Brain Res.* 1220 191–198 10.1016/j.brainres.2008.01.014 18299121

[B10] Chase W. G. Simon H. A. (1973). Perception in chess. *Cogn. Psychol.* 4 55–81 10.1016/0010-0285(73)90004-2

[B11] Chi M. T. Feltovich P. J. Glaser R. (1981). Categorization and representation of physics problems by experts and novices. *Cogn. Sci.* 5 121–152 10.1207/s15516709cog0502_2

[B12] Connors M. H. Campitelli G. (2014). Expertise and the representation of space. *Front. Psychol.* 5:270 10.3389/fpsyg.2014.00270 PMC3982051 24765081

[B13] Crump M. J. McDonnell J. V. Gureckis T. M. (2013). Evaluating Amazon’s Mechanical Turk as a tool for experimental behavioral research. *PLoS ONE* 8:e57410 10.1371/journal.pone.0057410 PMC3596391 23516406

[B14] De Leeuw J. R. (2014). jsPsych: a JavaScript library for creating behavioral experiments in a web browser. *Behav. Res. Methods* 10.3758/s13428-014-0458-y [Epub ahead of print] 24683129

[B15] Dror I. E. Cole S. A. (2010). The vision in “blind” justice: expert perception, judgment, and visual cognition in forensic pattern recognition. *Psychon. Bull. Rev.* 17 161–167 10.3758/PBR.17.2.161 20382914

[B16] Duchaine B. Nakayama K. (2006). The Cambridge Face Memory Test: results for neurologically intact individuals and an investigation of its validity using inverted face stimuli and prosopagnosic participants. *Neuropsychologia* 44 576–585 10.1016/j.neuropsychologia.2005.07.001 16169565

[B17] Ericsson K. A. (2006). “The influence of experience and deliberate practice on the development of superior expert performance,” in *The Cambridge Handbook of Expertise and Expert Performance* eds Ericsson K. A. Charness N. Feltovich P. Hoffman R. R. (Cambridge, UK:Cambridge University Press) 691–698 10.1017/CBO9780511816796.038

[B18] Ericsson K. A. (2009). “Enhancing the development of professional performance: implications from the study of deliberate practice,” in *The Development of Professional Expertise: Toward Measurement of Expert Performance and Design of Optimal Learning Environments* ed. Ericsson K. A. (New York, NY:Cambridge University Press) 412–425 10.1017/CBO9780511609817.022

[B19] Gauthier I. McGugin R. Richler J. J. Herzmann G. Speegle M. Van Gulick A. E. (2013). Experience with objects moderates the overlap between object and face recognition performance, suggesting a common ability. *J. Vis.* 13 982–982 10.1167/13.9.982 PMC4528484 24993021

[B20] Gauthier I. Skudlarski P. Gore J. C. Anderson A. W. (2000). Expertise for cars and birds recruits brain areas involved in face recognition. *Nat. Neurosci.* 3 191–197 10.1038/72140 10649576

[B21] Gauthier I. Tarr M. J. (1997). Becoming a “Greeble” expert: exploring mechanisms for face recognition. *Vision Res.* 37 1673–1682 10.1016/S0042-6989(96)00286-6 9231232

[B22] Gauthier I. Tarr M. J. (2002). Unraveling mechanisms for expert object recognition: bridging brain activity and behavior. *J. Exp. Psychol. Hum. Percept. Perform.* 28 431–446 10.1037//0096-1523.28.2.431 11999864

[B23] Gauthier I. Tarr M. J. Anderson A. W. Skudlarski P. Gore J. C. (1999). Activation of the middle fusiform “face area” increases with expertise in recognizing novel objects. *Nat. Neurosci.* 2 568–573 10.1038/9224 10448223

[B24] Gauthier I. Tarr M. J. Bub D. N. (eds) (2010). *Perceptual Expertise: Bridging Brain and Behavior*. New York, NY:Oxford University Press 10.1093/acprof:oso/9780195309607.001.0001

[B25] Gauthier I. Williams P. Tarr M. J. Tanaka J. (1998). Training “greeble” experts: a framework for studying expert object recognition processes. *Vision Res.* 38 2401–2428 10.1016/S0042-6989(97)00442-2 9798007

[B26] Gobet F. Charness N. (2006). “Expertise in chess,” in *The Cambridge Handbook of Expertise and Expert Performance* eds Ericsson K. A. Charness N. Feltovich P. Hoffman R. R. (Cambridge, UK:Cambridge University Press) 523–538 10.1017/CBO9780511816796.038

[B27] Goldstone R. L. (1998). Perceptual learning. *Annu. Rev. Psychol.* 49 585–612 10.1146/annurev.psych.49.1.585 9496632

[B28] Holt L. L. Lotto A. J. (2008). Speech perception within an auditory cognitive science framework. *Curr. Dir. Psychol. Sci.* 17 42–46 10.1111/j.1467-8721.2008.00545.x 19060961 PMC2593873

[B29] Johnson K. E. Mervis C. B. (1997). Effects of varying levels of expertise on the basic level of categorization. *J. Exp. Psychol. Gen.* 126 248–277 10.1037/0096-3445.126.3.248 9281832

[B30] Jolicoeur P. Gluck M. A. Kosslyn S. M. (1984). Pictures and names: making the connection. *Cogn. Psychol.* 16 243–275 10.1016/0010-0285(84)90009-4 6734136

[B31] Kaufman K. (1999). *A Peterson Field Guide to Advanced Birding: Birding Challenges and How to Approach Them.* Boston, MA:Houghton Miﬄin Harcourt

[B32] Kaufman K. (2011). *Kaufman Field Guide to Advanced Birding: Understanding What You See and Hear*. New York, NY:Houghton Miﬄin Harcourt

[B33] La Rouche G. P. (2006). “Birding in the united states: a demographic and economic analysis,” in *Waterbirds Around the World,* eds Boere G. C. Galbraith C. A. Stroud D. A. (Edinburgh, UK:The Stationery Office) 841–846

[B34] Leone M. J. Fernandez Slezak D. Cecchi G. A. Sigman M. (2014). The geometry of expertise. *Front. Psychol.* 5:47 10.3389/fpsyg.2014.00047 PMC3913042 24550869

[B35] Mack M. L. Palmeri T. J. (2011). The timing of visual object categorization. *Front. Psychol.* 2:165 10.3389/fpsyg.2011.00165 PMC3139955 21811480

[B36] Mack M. L. Wong A. C.-N. Gauthier I. Tanaka J. W. Palmeri T. J. (2007). “Unraveling the time-course of perceptual categorization: does fastest mean first?” in *Proceedings of the 29th Annual Conference of the Cognitive Science Society* Mahwah, NJ 1253–1258

[B37] Mack M. L. Wong A. C.-N. Gauthier I. Tanaka J. W. Palmeri T. J. (2009). Time course of visual object categorization: fastest does not necessarily mean first. *Vision Res.* 49 1961–1968 10.1016/j.visres.2009.05.005 19460401

[B38] McCandliss B. D. Cohen L. Dehaene S. (2003). The visual word form area: expertise for reading in the fusiform gyrus. *Trends Cogn. Sci.* 7 293–299 10.1016/S1364-6613(03)00134-7 12860187

[B39] McGugin R. W. Richler J. J. Herzmann G. Speegle M. Gauthier I. (2012). The vanderbilt expertise test reveals domain-general and domain-specific sex effects in object recognition. *Vision Res.* 69 10–22 10.1016/j.visres.2012.07.014 22877929 PMC3513270

[B40] McGugin R. W. Van Gulick A. E. Tamber-Rosenau B. J. Ross D. A. Gauthier I. (2014). Expertise effects in face-selective areas are robust to clutter and diverted attention, but not to competition. *Cereb. Cortex* 10.1093/cercor/bhu060 [Epub ahead of print] PMC4537424 24682187

[B41] Mitchell C. Hall G. (2014). Can theories of animal discrimination explain perceptual learning in humans? *Psychol. Bull.* 140 283–307 10.1037/a0032765 23647232

[B42] National Research Council of the National Academy of Sciences. (2009). *Strengthening Forensic Science in the United States: A Path Forward*. Washington, DC:National Academies Press

[B43] Palmeri T. J. (1997). Exemplar similarity and the development of automaticity. *J. Exp. Psychol. Learn. Mem. Cogn.* 23 324–54 10.1037//0278-7393.23.2.324 9080007

[B44] Palmeri T. J. Cottrell G. W. (2009). “Modeling Perceptual Expertise,” in *Perceptual Expertise: Bridging Brain and Behavior,* eds Bub D. N. Tarr M. J. Gauthier I. (New York, NY:Oxford University Press) 197–245 10.1093/acprof:oso/9780195309607.003.0008

[B45] Palmeri T. J. Gauthier I. (2004). Visual object understanding. *Nat. Rev. Neurosci.* 5 291–303 10.1038/nrn1364 15034554

[B46] Palmeri T. J. Wong A. C.-N. Gauthier I. (2004). Computational approaches to the development of perceptual expertise. *Trends Cogn. Sci.* 8 378–386 10.1016/j.tics.2004.06.001 15335465

[B47] Reimers S. Stewart N. (2007). Adobe Flash as a medium for online experimentation: A test of reaction time measurement capabilities. *Behav. Res. Methods* 39 365–370 10.3758/BF03193004 17958146

[B48] Reuter E. M. Voelcker-Rehage C. Vieluf S. Godde B. (2012). Touch perception throughout working life: Effects of age and expertise. *Exp. Brain Res.* 216 287–297 10.1007/s00221-011-2931-5 22080104

[B49] Richler J. J. Palmeri T. J. (2014). Visual category learning. *WIREs Cogn. Sci.* 5 75–94 10.1002/wcs.1268 26304297

[B50] Richler J. J. Wong Y. K. Gauthier I. (2011). Perceptual expertise as a shift from strategic interference to automatic holistic processing. *Curr. Dir. Psychol. Sci.* 20 129–134 10.1177/0963721411402472 21643512 PMC3104280

[B51] Rosch E. Mervis C. B. Gray W. D. Johnson D. M. Boyes-Braem P. (1976). Basic objects in natural categories. *Cogn. Psychol.* 8 382–439 10.1016/0010-0285(76)90013-X

[B52] Simcox T. Fiez J. A. (2014). Collecting response times using Amazon Mechanical Turk and Adobe Flash. *Behav. Res. Methods* 46 95–111 10.3758/s13428-013-0345-y 23670340 PMC5283577

[B53] Tanaka J. W. Curran T. Sheinberg D. L. (2005). The training and transfer of real-world perceptual expertise. *Psychol. Sci.* 16 145–151 10.1111/j.0956-7976.2005.00795.x 15686581

[B54] Tanaka J. W. Taylor M. (1991). Object categories and expertise: Is the basic level in the eye of the beholder? *Cogn. Psychol.* 23 457–482 10.1016/0010-0285(91)90016-H

[B55] Tanaka J. W. Wolf J. M. Klaiman C. Koenig K. Cockburn J. Herlihy L. (2010). Using computerized games to teach face recognition skills to children with autism spectrum disorder: the Let’s Face It! program. *J. Child Psychol. Psychiatry* 51 944–952 10.1111/j.1469-7610.2010.02258.x 20646129

[B56] Van Gulick A. E. (2014). *Measurement of Semantic Knowledge and its Contribution to Object Recognition Performance.* Doctoral Dissertation, Vanderbilt University, Nashville, TN.

[B57] Wilmer J. B. Germine L. Chabris C. F. Chatterjee G. Williams M. Loken E. (2010). Human face recognition ability is specific and highly heritable. *Proc. Natl. Acad. Sci. U.S.A.* 107 5238–5241 10.1073/pnas.0913053107 20176944 PMC2841913

[B58] Wolfe J. M. Brunelli D. N. Rubinstein J. Horowitz T. S. (2013). Prevalence effects in newly trained airport checkpoint screeners: trained observers miss rare targets, too. *J. Vis.* 13 1–9 10.1167/13.3.33 PMC3848386 24297778

[B59] Wong A. C.-N. Palmeri T. J. Gauthier I. (2009a). Conditions for facelike expertise with objects: becoming a ziggerin expert—but which type? *Psychol. Sci.* 20 1108–1117 10.1111/j.1467-9280.2009.02430.x 19694980 PMC2919853

[B60] Wong A. C.-N. Palmeri T. J. Rogers B. P. Gore J. C. Gauthier I. (2009b). Beyond shape: how you learn about objects affects how they are represented in visual cortex. *PLoS ONE* 4:e8405 10.1371/journal.pone.0008405 PMC2794531 20027229

